# The Role of Robotic Visceral Surgery in Patients with Adhesions: A Systematic Review and Meta-Analysis

**DOI:** 10.3390/jpm12020307

**Published:** 2022-02-18

**Authors:** Marco Milone, Michele Manigrasso, Pietro Anoldo, Anna D’Amore, Ugo Elmore, Mariano Cesare Giglio, Gianluca Rompianesi, Sara Vertaldi, Roberto Ivan Troisi, Nader K. Francis, Giovanni Domenico De Palma

**Affiliations:** 1 Department of Clinical Medicine and Surgery, University of Naples “Federico II”, 80131 Naples, Italy; anna.damore1993@libero.it (A.D.); mariano.giglio@hotmail.it (M.C.G.); gianluca.rompianesi@unina.it (G.R.); vertaldisara@gmail.com (S.V.); roberto.troisi@unina.it (R.I.T.); giovanni.depalma@unina.it (G.D.D.P.); 2 Department of Advanced Biomedical Sciences, University of Naples “Federico II”, 80131 Naples, Italy; michele.manigrasso@unina.it (M.M.); pietro.anoldo@gmail.com (P.A.); 3 Department of Surgery, San Raffaele Hospital and San Raffaele Vita-Salute University, 20132 Milan, Italy; elmore.ugo@hsr.it; 4 Yeovil District Hospital, Somerset BA21 4AT, UK; nader.francis@ydh.nhs.uk

**Keywords:** conversion, abdominal adhesions, laparoscopic surgery, robotic surgery

## Abstract

Abdominal adhesions are a risk factor for conversion to open surgery. An advantage of robotic surgery is the lower rate of unplanned conversions. A systematic review was conducted using the terms “laparoscopic” and “robotic”. Inclusion criteria were: comparative studies evaluating patients undergoing laparoscopic and robotic surgery; reporting data on conversion to open surgery for each group due to adhesions and studies including at least five patients in each group. The main outcomes were the conversion rates due to adhesions and surgeons’ expertise (novice vs. expert). The meta-analysis included 70 studies from different surgical specialities with 14,329 procedures (6472 robotic and 7857 laparoscopic). The robotic approach was associated with a reduced risk of conversion (OR 1.53, 95% CI 1.12–2.10, *p* = 0.007). The analysis of the procedures performed by “expert surgeons” showed a statistically significant difference in favour of robotic surgery (OR 1.48, 95% CI 1.03–2.12, *p* = 0.03). A reduced conversion rate due to adhesions with the robotic approach was observed in patients undergoing colorectal cancer surgery (OR 2.62, 95% CI 1.20–5.72, *p* = 0.02). The robotic approach could be a valid option in patients with abdominal adhesions, especially in the subgroup of those undergoing colorectal cancer resection performed by expert surgeons.

## 1. Introduction

Robotic surgery was introduced in the early 2000s to overcome some technical limitations of conventional laparoscopic surgery. However, even if some benefits of the robotic approach over laparoscopy have been described [1,2,3,4,5], it is currently considered the gold standard treatment only for radical prostatectomy [6].

Specific interventions that could benefit from the robotic approach are yet to be identified. It is worth mentioning that one of the most extensively reported advantages of robotic surgery is the lower rate of unplanned open conversions [7,8,9,10,11,12,13,14]. Conversion to open surgery can be multifactorial, and when all causes of conversion were examined in ROLARR Randomized Controlled Clinical Trial (RCT), no difference was found between robotic and laparoscopic techniques during rectal cancer surgery [15].

Intra-abdominal adhesions due to prior abdominal surgery are a common and well-recognised risk factor for conversion [16,17,18], and it is not known whether the robotic approach could allow a lower conversion rate than laparoscopy in patients with adhesions. The rationale lies in the potential technical advantages of robotic surgery—magnified 3D vision with a more stable operative field, preservation of natural eye-hand-instrument alignment, precisely controlled EndoWrist instruments with better ergonomics and reduced physiologic tremor—heightened in case of distortion of the normal abdominal anatomy related to adhesions, which makes the visualisation more difficult and increases the difficulty of surgical procedure.

Since the indications for the robotic technique outside prostatectomy are far from being established by high levels of evidence, a meta-analysis of the available literature addressing pertinent questions related to the possible benefits of the robotic approach over laparoscopy is required to guide the expansion of the application of the robotic techniques. The aim of this study was to systematically review the literature and pool the evidence in order to evaluate and compare the adhesion-related conversions to open surgery are in patients undergoing robotic and laparoscopic surgery across all specialities.

## 2. Materials and Methods

### 2.1. Literature Search and Study Selection

To identify all available studies, an electronic search of Cochrane Library (including the Cochrane Central Register of Controlled Trials), EMBASE, PubMed, SCOPUS and Web of Science was conducted according to PRISMA (Preferred Reporting Items for Systematic reviews and Meta-Analyses) guidelines [19]. This systematic review was performed following the meta-analysis of observational studies epidemiology (MOOSE) guidelines [20].

The search terms “laparoscopic” and “robotic” were used. The search was limited to studies regarding humans and published in English between June 1993 and March 2020.

Inclusion criteria were as follows: (1) comparative studies evaluating patients undergoing laparoscopic and robotic surgery; (2) studies reporting data on conversion to open surgery for each group due to adhesions; and (3) studies including at least 5 patients in each group, to minimise the imprecision associated with very small populations. Indexed abstracts of posters and podium presentations at international meetings were not included. Systematic reviews and meta-analyses were consulted to find additional studies of interest. Reference lists of the selected studies were screened to find additional studies of interest. If the same author or institution published overlapping series in different articles, only the most recent study was included. Two reviewers (Mi.Ma. and S.V.) independently assessed the reports for eligibility at the title and abstract level. In case of discrepancies, a third author (M.M.) was consulted, and an agreement was reached by consensus.

### 2.2. Data Extraction and Quality Assessment of Included Studies

The following data were extracted from each included study: first author, year of publication, study design, propensity score analysis, surgical field, diagnosis, type of intervention, total number of patients, number of patients undergoing laparoscopic and robotic surgery, and number of conversions related to intraoperative adhesions. Although widely reported by surgical studies, the definition of conversion within the literature varies [21]; therefore, we searched for this information in all the included studies. Surgeons’ expertise (classified as novice vs. expert) has been described in many of the included studies, even if only a few studies reported the number of procedures performed by the surgeons. None of the studies provided an exact definition of the various steps of the surgical procedure. Thus, the criteria to define expertise remains heterogeneous.

Furthermore, attempts to examine the quality assurance of surgical techniques of the studies according to Foster JD et al. [22] was performed for the assessment of surgeon-dependent performance bias.

The following patients’ characteristics were extracted and registered: gender, mean age, mean BMI, American Society of Anesthesiologists (ASA) score and previous abdominal surgery.

Study quality assessment for non-randomised clinical trial was performed using the Newcastle Ottawa Scale (NOS) [23]. This scoring system encompasses three major domains (selection, comparability and exposure), with a resulting score that varies between 0 (low quality) and 9 (high quality). In the case of randomised controlled trial (RCTs), the risk of bias was evaluated according to the Cochrane Collaboration Tool for assessing the risk of bias [24]. According to this scoring system, seven domains were evaluated as “Low risk of bias” or “High risk of bias” or “Unclear” according to reporting on sequence generation, allocation concealment, blinding of participants, blinding of outcome assessment, incomplete outcome data, selective outcome reporting and other potential threats to validity. The results of the quality assessment are reported in Table 1.

### 2.3. Statistical Analysis

Statistical analysis was performed using RevMan (Version 5.4, Copenhagen: The Nordic Cochrane Centre, The Cochrane Collaboration, 2020).

The primary outcome of this study was the open conversion rate to open surgery due to adhesions. The odds ratio (OR) along with 95% confidence interval (CI) was used as an effect estimate for dichotomous outcomes, with OR values < 1 indicating fewer events in the robotic group. In the case of zero events, a 0.5 correction was added to incorporate all available data in the meta-analysis and to maintain analytic consistency [94]. When studies provided only means for continuous variables and sample size of the trial, a standard deviation was imputed, according to Furukawa et al. [95]. The summary estimate was computed according to the random effect model described by DerSimonian and Laird [96]. A conservative random effect model was chosen a priori in consideration of foreseen heterogeneity among the studies, which were from different surgical fields. The heterogeneity among studies was tested by Q statistic and quantified by I2 statistic, with I2 values < 25%, between 25 and 50%, and >50% indicating respectively low, moderate, and high heterogeneity [97].

With the aim to assess if that differences among included studies may be affected by demographic (gender, age and BMI) and clinical variables (ASA Score and previous abdominal surgery), we planned to perform meta-regression analyses in case of the significance of the meta-analysis after implementing a regression model with incidence of the main outcome as dependent variable (y) and the above-mentioned covariates as independent variables (x). Meta-regression analyses were performed with Comprehensive Meta-analysis (Version 2.2, Biostat Inc., Englewood, NJ, USA, 2005), provided by Biostat Inc. [98].

The presence of publication bias was investigated through a funnel plot where the summary estimate of each study (OR) was plotted against a measure of study precision (standard error). In addition to visual inspection, funnel plot symmetry was tested using Egger’s linear regression method [99]. *p* values < 0.05 were considered statistically significant.

Furthermore, different subgroups analyses, including studies about each surgical field (colorectal, oesophagogastric, hepatobiliary, pancreatic, endocrine, urologic and gynaecologic surgery) and the surgeons’ expertise (novice and expert) were performed. Furthermore, in case of a statistically significant difference in any of the above-mentioned surgical fields, further analyses were performed to understand if the significance was present in the case of benign and or malignant disease.

## 3. Results

### 3.1. Study Selection

The electronic search provided a total of 49,891 results. After the removal of duplicates, 10,489 studies underwent screening on the basis of title. Of the 4050 full-text articles assessed for eligibility, 3978 studies were excluded for several reasons: 431 were not published in the English language, 444 were case reports, 2179 were reviews, 535 were off-topic after scanning abstract, and for 391, data were not available. At the end of the selection process, 70 studies were included in the meta-analysis [13,25,26,27,28,29,30,31,32,33,34,35,36,37,38,39,40,41,42,43,44,45,46,47,48,49,50,51,52,53,54,55,56,57,58,59,60,61,62,63,64,65,66,67,68,69,70,71,72,73,74,75,76,77,78,79,80,81,82,83,84,85,86,87,88,89,90,91,92,93].

### 3.2. Study Characteristics

The selected studies included a total of 14,329 patients, of whom 6472 underwent robotic surgery and 7857 laparoscopic surgery. Fifty-one studies were retrospective [13,25,26,27,32,33,37,39,40,41,42,43,44,45,46,47,48,49,51,52,53,56,57,58,63,64,65,66,67,68,69,71,73,74,75,76,77,78,79,80,81,82,83,84,85,86,87,88,89,90,92,93], eighteen were prospective [28,29,30,31,34,35,36,38,50,54,55,59,60,61,62,65,72,91], and there was only one randomised controlled trial [70]. Studies were from different fields of surgery, including colorectal (*n* = 19), oesophagogastric (*n* = 10), hepatobiliary (*n* = 5), pancreatic (*n* = 6), gynaecologic (*n* = 19), urologic (*n* = 5), endocrine (*n* = 3) vascular (*n* = 1), abdominal wall (*n* = 1) and splenic surgery (*n* = 1). In six studies, robotic surgery was performed by early surgeons, and by expert surgeons in other 47 studies. The other 17 studies did not provide these data. The characteristics of the included studies are reported in Table 2.

### 3.3. Quality Assessment of Studies and Performance

All studies had NOS quality scores greater than 6, indicating that all these studies had a high methodological quality. Specifically, twenty-one studies had NOS quality score = 9; thirty studies had NOS quality score = 8; eighteen studies had NOS quality score = 7. The NOS quality score is shown in Table 1. The only randomised controlled trial (RCT) showed a low risk of bias.

Among the expert surgeons, none of the included studies reported on the quality assurance of surgical technique as described by Foster et al. [22]. Thus, it was not possible to perform further analyses on the quality of surgical performance among expert or early surgeons.

### 3.4. Conversion to Open Surgery Due to Adhesions

**Figure 1:**
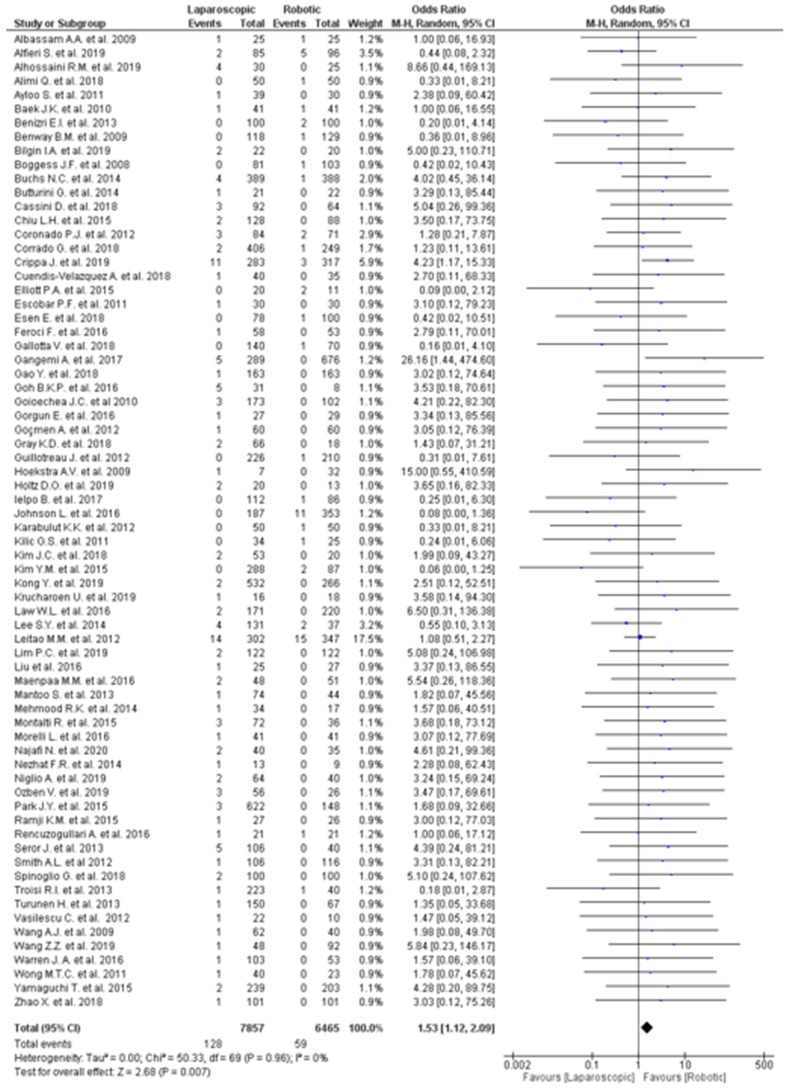
Meta-analysis of the included studies on conversion due to adhesions.

Seventy studies provided data about the conversion to open surgery due to anastomotic adhesions [13,25,26,27,28,29,30,31,32,33,34,35,36,37,38,39,40,41,42,43,44,45,46,47,48,49,50,51,52,53,54,55,56,57,58,59,60,61,62,63,64,65,66,67,68,69,70,71,72,73,74,75,76,77,78,79,80,81,82,83,84,85,86,87,88,89,90,91,92,93], even if only nine of them [13,35,41,43,45,46,50,78,84] reported the definition of conversion. The robotic approach was associated with a reduced risk of conversion (OR 1.53, 95% CI 1.12–2.10, *p* = 0.007, Figure 1), with consistent results across all the 70 studies since no heterogeneity was observed (I^2^ = 0%, *p* = 0.95).

**Figure 2:**
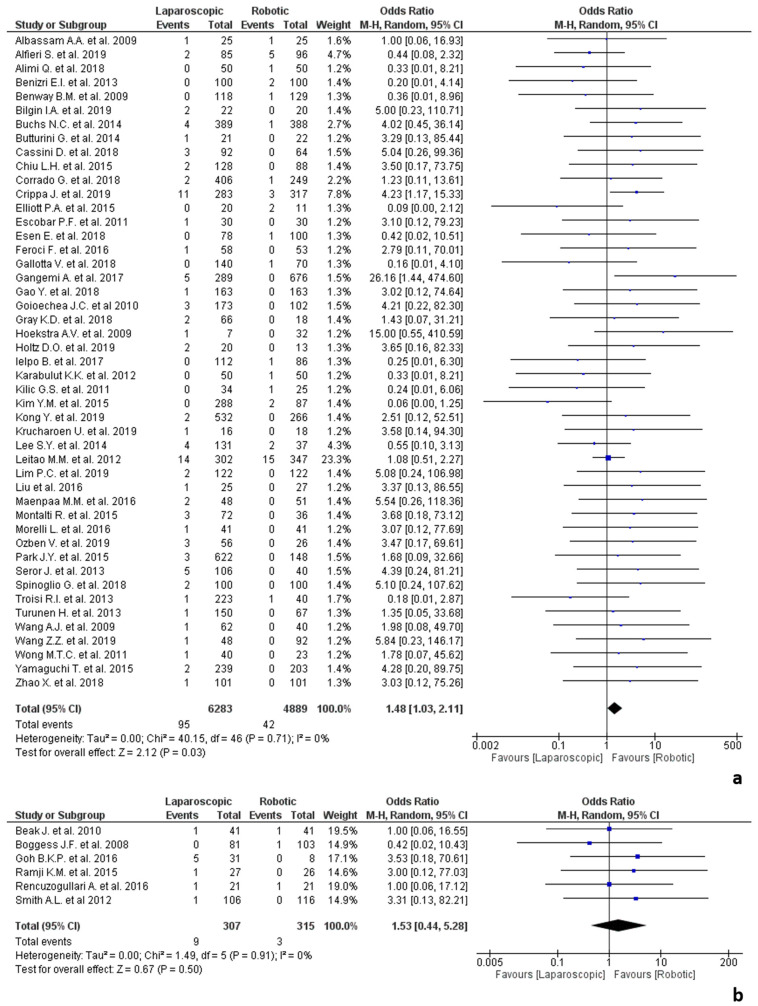
Conversions due to adhesions according to surgeons’ expertise: (**a**) in procedures performed by expert surgeons; (**b**) in procedures performed by “early” surgeons.

Regarding surgeons’ expertise, 47 studies classified surgeons as “expert” [25,26,28,31,32,33,35,36,37,40,41,43,44,45,46,47,48,49,51,53,55,56,57,59,60,62,63,64,66,67,68,69,70,73,74,78,79,82,84,85,86,88,89,91,92,93] and 6 studies as “novice” [13,30,34,80,81,83]. The analysis of the procedures performed by expert surgeons involved 11,172 procedures, of which 6283 laparoscopic and 4889 robotic and showed a statistically significant difference in favour of robotic surgery (OR 1.48, 95% CI 1.03–2.12, *p* = 0.03), with no heterogeneity among the studies (I^2^ = 0%, *p* = 0.71). The analysis of the procedures performed by “novice” surgeons involved 622 procedures, of which 307 laparoscopic and 315 robotic and showed no significant difference between the two groups (OR 1.53, 95% CI 0.44–5.28, *p* = 0.50), without any heterogeneity among the studies (I^2^ = 0%, *p* = 0.91). Data on surgeons’ expertise are shown in Figure 2.

Our meta-regression analysis showed that no demographic or clinical outcomes significantly impacted conversion, as shown in Table 3.

### 3.5. Subgroup Analysis

#### 3.5.1. Colorectal Surgery

**Figure 3:**
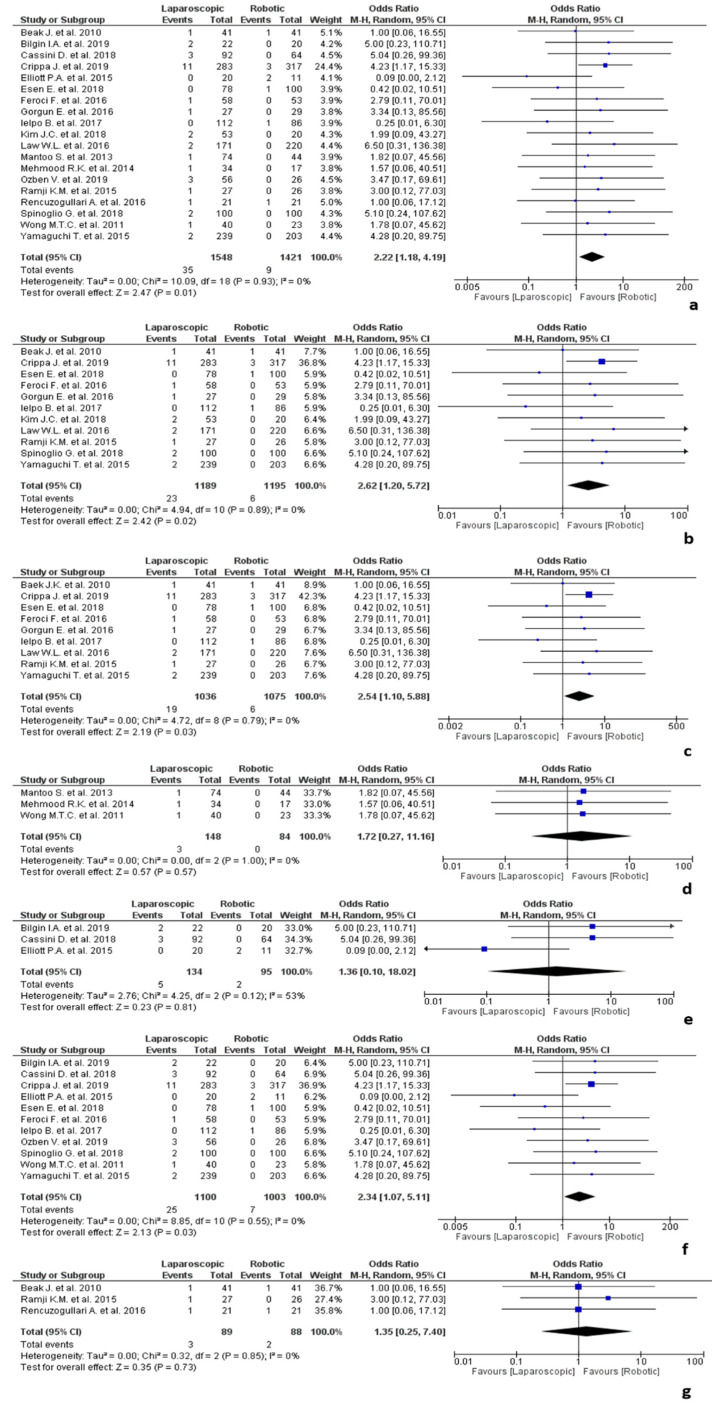
Results of the studies about colorectal surgery: (**a**) conversion due to adhesions in colorectal surgery; (**b**) conversion due to adhesions in colorectal cancer surgery; (**c**) conversion due to adhesions in rectal cancer surgery; (**d**) conversion due to adhesions in colorectal surgery for rectal prolapse/rectocele; (**e**) conversion due to adhesions in colorectal surgery for diverticular disease; (**f**) conversion due to adhesions in colorectal surgery performed by expert surgeons; (**g**) conversion due to adhesions in colorectal surgery performed by “early” surgeons.

The results of the studies about colorectal surgery are shown in Figure 3. Nineteen studies [30,33,37,41,43,45,46,52,57,61,65,71,72,78,80,81,84,91,92] included in the final analysis were including colorectal surgery cases and involved 2969 procedures, of which 1548 laparoscopic and 1421 robotic. Of the included studies, eleven were on colorectal cancer [30,41,45,46,52,57,61,65,80,84,92], three on rectocele or rectal prolapse [71,72,91] and three on diverticular disease patients [33,37,43]. Ozben et al. [78] described surgical procedures related to both benign and malign diseases. Rencuzogullari et al. [81] was the only one to report surgical proctectomy performed for IBD, so it was excluded from the subgroup analysis.

In the overall colorectal surgery analysis, a significant difference in terms of conversion rate related to adhesions was observed between the two groups in favour of robotics (OR 2.22, 95% CI 1.18–4.19, *p* = 0.01), with no heterogeneity among the included studies (I^2^ = 0%, *p* = 0.93).

Meta-regression analysis showed that none of the demographic and clinical parameters (gender, age, BMI, ASA and tumoural stage) significantly impacted the conversion rate due to adhesions, with the exception of “previous abdominal surgery” (*p* = 0.03).

In a further analysis about colorectal cancer the significance was confirmed (OR 2.62, 95% CI 1.20–5.72, *p* = 0.02), with no heterogeneity among the included studies (I^2^ = 0%, *p* = 0.89). Even including only studies about rectal cancer [30,41,45,46,52,57,61,65,80,84,92], the significance was confirmed (OR 2.54, 95% CI 1.10–5.88, *p* = 0.03), with no heterogeneity among the included studies (I^2^ = 0%, *p* = 0.79).

Meta-regression analysis on colorectal cancer showed that none of the demographic or clinical parameters significantly impacted the analysed outcome.

No statistically significant differences in terms of conversion rate due to adhesions were observed between robotics and laparoscopy in the studies about rectocele/rectal prolapse [72,73,91] and diverticular disease [33,37,43] (OR 1.72, 95% CI 0.27–11.16, *p* = 0.57 and OR 1.36, 95% CI 0.10–18.02, *p* = 0.81, respectively), with no significant heterogeneity among the studies (I^2^ = 0%, *p* = 1.00 and I^2^ = 53%, *p* = 0.12, respectively).

Within the colorectal surgery studies, surgeons were classified as “expert” in eleven studies [33,37,41,43,45,46,57,78,84,91,92] and as “novice” in other three studies [30,80,81]. Five studies did not provide these data [52,61,65,71,72]. The analysis about expertise in colorectal surgeries showed that a significant difference in terms of conversion rate related to adhesions was found in colorectal surgery performed by expert surgeons in favour of robotic approach (OR 2.34, 95% CI 1.07–5.11, *p* = 0.03), while no statistically significant differences were observed among colorectal (OR 1.35, 95% CI 0.25–7.40, *p* = 0.73) “novice” surgeons.

#### 3.5.2. Oesophagogastric Surgery

**Figure 4:**
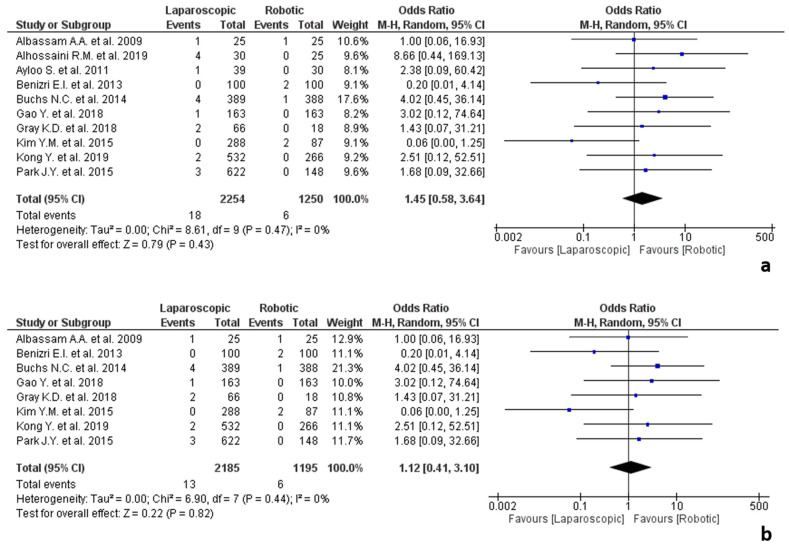
Results of the studies about oesophagogastric surgery: (**a**) conversion due to adhesions in oesophago-gastric surgery; (**b**) conversion due to adhesions in oesophagogastric surgery performed by expert surgeons.

The results of the studies about oesophagogastric surgery are shown in Figure 4. Ten studies addressed oesophagogastric surgery [25,27,29,31,35,49,53,62,63,79], involving 3504 procedures, 2254 of which were laparoscopic and 1250 robotic. Of the included studies, five were about gastric cancer [27,49,62,63,79], four about morbid obesity [29,31,35,53] (two about Roux-en-Y gastric bypass [31,35], one about sleeve gastrectomy [29] and one about different surgical procedures for bariatric revisional surgery [53]) and one on Nissen fundoplication for reflux disease [25]. No statistically significant differences were found between the two groups in terms of conversion rate related to adhesions (OR 1.45, 95% CI 0.58–3.64, *p* = 0.43), with no heterogeneity among the included studies (I^2^ = 0%, *p* = 0.47).

Surgeons’ expertise was reported by eight studies [25,31,35,49,53,62,63,79], that classified surgeons as experts. The other two studies did not report on these data [27,29]. The meta-analysis about surgeons’ expertise showed that no statistically significant differences were found among the expert surgeon between robotic and laparoscopic conversion rate (OR 1.12, 95% CI 0.41–3.10, *p* = 0.82), with no heterogeneity among the studies (I^2^ = 0%, *p* = 0.44). The analysis about “novice” surgeons was not possible because none of the included studies reported these data.

#### 3.5.3. Gynaecologic Surgery

**Figure 5:**
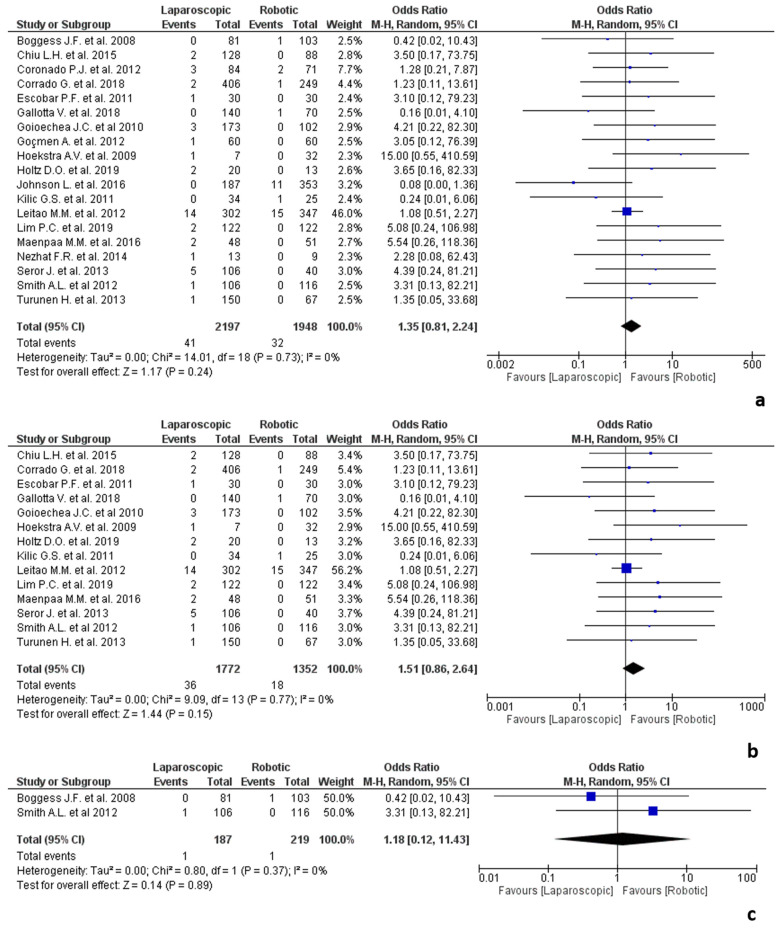
Results of the studies about gynaecologic surgery: (**a**) conversion due to adhesions in gynaecologic surgery; (**b**) conversion due to adhesions in gynaecologic surgery performed by expert surgeons; (**c**) conversion due to adhesions in gynaecologic surgery performed by “early” surgeons.

The results of the studies about gynaecologic surgery are shown in Figure 5. Nineteen studies about gynaecologic surgery were included in the meta-analysis [34,38,39,40,44,47,50,51,55,56,58,60,67,68,70,76,82,83,86]. Of the included studies, 18 reported data on hysterectomies performed for benign [50,60] or malignant conditions [34,39,40,44,47,51,55,56,58,67,68,70,82,83,86] or the combination of malignancy and benign diseases [38] and one about salpingo-oophorectomy due to early ovarian cancer [76]. The included studies involved 3124 procedures, of which 1772 were laparoscopic and 1352 robotic, with no statistically significant difference between the two groups (OR 1.36, 95% CI 0.82–2.25, *p* = 0.24) in terms of conversion rate related to adhesions and no heterogeneity among the included studies (I^2^ = 0%, *p* = 0.73).

Fourteen studies classified the surgeons as “experts” [40,44,47,50,51,55,56,58,60,67,68,70,82,86], two as “novice” [34,83] and four did not report on these data [38,39,50,76]. No significant difference in terms of conversion rate related to adhesions was found between the two groups in the procedures performed by both expert or novice surgeons (OR 1.52, 95% CI 0.87–2.65, *p* = 0.14 and OR 1.18, 95% CI 0.12–11.43, *p* = 0.89), with no heterogeneity among the studies (I^2^ = 0%, *p* = 0.77 and I^2^ = 0%, *p* = 0.37).

#### 3.5.4. Hepatobiliary Surgery

**Figure 6:**
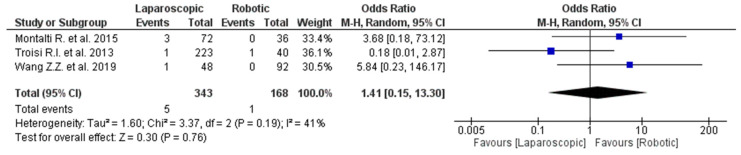
Conversion due to adhesions in hepatobiliary surgery.

The results of the five hepatobiliary surgery studies are shown in Figure 6 [42,48,73,85,89]. Of the included studies, three [73,85,89] included 511 liver resections, 343 laparoscopic and 168 robotic, and all the studies classified surgeons as experts.

Cuendis-Velazquez A. et al. [41] and Gangemi et al. [48] reported hepaticojejunostomy performed for bile duct injury and cholecystectomy, respectively, so they were excluded from our analysis.

No differences were found in terms of conversion due to adhesions between the two groups (OR 1.41, 95% CI 0.15–13.30, *p* = 0.76), without a significant heterogeneity among the included studies (I^2^ = 41%, *p* = 0.76).

#### 3.5.5. Pancreatic Surgery

**Figure 7:**
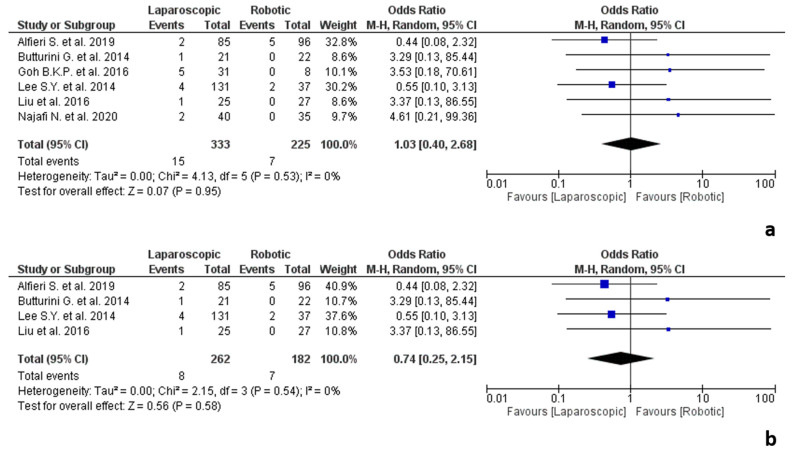
Results of the studies about pancreatic surgery: (**a**) conversion due to adhesions in pancreatic surgery; (**b**) conversion due to adhesions in pancreatic surgery performed by expert surgeons.

The results of the studies about pancreatic surgery are shown in Figure 7. Six studies [13,26,36,66,69,75] reporting data about conversion due to adhesions in pancreatic surgery were included in the meta-analysis, involving 558 procedures, 333 laparoscopic and 225 robotic. Of the included studies, four [13,26,36,66] reported data on distal pancreatectomies for pancreatic tumours [13,36,66] or neuroendocrine tumours (pNETs) [26], one about pancreaticoduodenectomies for periampullary neoplasms [69] and one about distal pancreatectomies or pancreatic enucleations for benign and borderline tumours [75]. The analysis showed no statistically significant difference in terms of conversion rate related to adhesions between the two groups (OR 1.03, 95% CI 0.40–2.68, *p* = 0.95), with no heterogeneity among the studies (I^2^ = 0%, *p* = 0.53).

Surgeons were classified as “expert” in four studies [26,36,66,69] and as “novice” in one study [13], while one study [75] did not report on these data.

In the case of surgery performed by expert surgeons, the analysis showed no significant differences in terms of conversion due to adhesions between the two groups (OR 0.74, 95% CI 0.25–2.15, *p* = 0.58), with no heterogeneity among the studies (I^2^ = 0%, *p* = 0.54).

#### 3.5.6. Urologic Surgery

**Figure 8:**
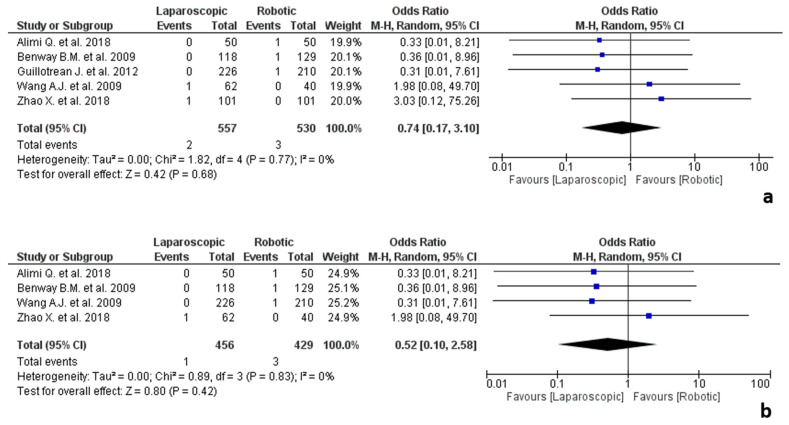
Results of the studies about urologic surgery: (**a**) conversion due to adhesions in urologic surgery; (**b**) conversion due to adhesions in urologic surgery performed by expert surgeons.

The results of the studies about urologic surgery are shown in Figure 8. Five studies [28,32,54,88,93] included partial nephrectomies [28,32,54,88] for renal cancer or simple enucleation with single layer renorrhaphy for localized renal tumours [93] were included in the analysis, involving 1087 procedures, 557 laparoscopic and 530 robotic.

No statistical difference was found in the two groups in terms of conversion due to adhesions (OR 0.74, 95% CI 0.17–3.10, *p* = 0.68), with no heterogeneity among the studies (I^2^ = 0%, *p* = 0.77).

Four studies [28,32,88,93] classified surgeons as experts, while one study [54] did not report on these data.

The analysis of the studies about expert surgeons showed no significant differences between the two groups (OR 0.92, 95% CI 0.18–4.58, *p* = 0.92), with no heterogeneity among the studies (I^2^ = 0%, *p* = 0.83).

#### 3.5.7. Endocrine Surgery

**Figure 9:**
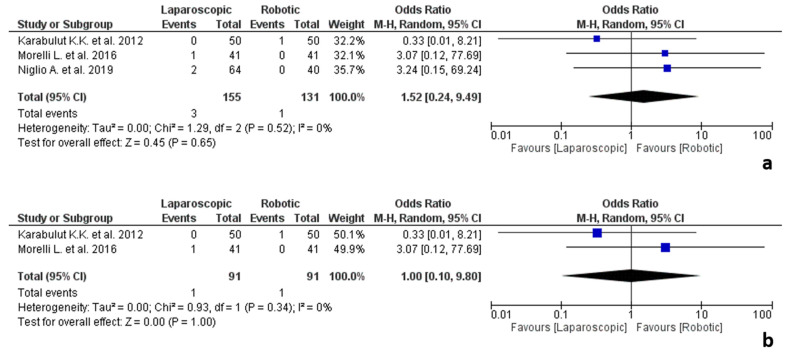
Results of the studies about endocrine surgery: (**a**) conversion due to adhesions in endocrine surgery; (**b**) conversion due to adhesions in endocrine surgery performed by expert surgeons.

The results of the endocrine surgery studies are shown in Figure 9. Three studies [59,74,77] that addressed adrenalectomies for adrenal cancer were included in the analysis, involving 286 procedures, 155 laparoscopic and 131 robotic.

No significant difference was found between the two groups in terms of conversion due to adhesions (OR 1.52, 95% CI 0.24–9.49, *p* = 0.65), with no heterogeneity among the studies (I^2^ = 0%, *p* = 0.52).

Surgeons’ expertise was reported by two studies [59,74], classifying surgeons as experts. One study [77] did not report on these data.

Analysis of the studies about expert surgeons showed no significant differences between the two groups (OR 1.00, 95% CI 0.10–9.80, *p* = 1.00), with no heterogeneity among the studies (I^2^ = 0%, *p* = 0.34).

#### 3.5.8. Other Surgical Fields

Khrucharoen et al. [64] described the median arcuate ligament (MAL) release for median arcuate ligament syndrome. Vasilescu et al. [87] reported splenectomy for hereditary spherocytosis. Warren et al. [90] described ventral hernia repair. These were individual studies for each respective surgical field, so it was not possible to perform a meta-analysis.

### 3.6. Publication Bias

Visual inspection of the funnel plot (Figure S1) showed symmetry, which was confirmed by Egger’s linear regression test (*p* = 0.12), indicating no publication bias. In the subgroup analyses, a symmetrical distribution of the studies was observed in all surgical fields except from pancreatic surgery, in which the visual inspection of the funnel plot suggested an asymmetric distribution of studies around the mean and the Egger’s test confirmed a significant publication bias (*p* = 0.0029).

## 4. Discussion

Since its introduction in the early 1990s, laparoscopic surgery has become the gold standard treatment of many benign and malignant conditions [100,101,102,103].

The advantages of a minimally invasive approach over an open approach are well proven [6,104], but laparoscopic surgery is technically challenging with a long learning curve.

Robotic surgery was introduced in the early 2000s to overcome these challenges of laparoscopic surgery, but to date, it is considered the gold standard treatment only for radical prostatectomy [6].

The efficacy and the feasibility of the robotic technique have been shown in various procedures across many surgical fields and demonstrate some benefits over the laparoscopic approach [3,4,105,106,107,108,109,110].

One of the reported benefits of robotic surgery is the lower rate of unplanned conversions to open surgery compared to laparoscopy [8,9,10,11,12,13,14,110]. This was, however, not supported by the results of an RCT on rectal cancer surgery and comparing conversions for all causes in robotic and laparoscopic procedures [15]. A cause–effect analysis is required to specifically target conversions related to adhesions and appraise the true impact of the robotic technique in comparison to laparoscopy in order to support the adoption of the robotic technique across all surgical fields.

By pooling together 14,329 patients, 6472 of whom were undergoing robotic surgery and 7857 laparoscopic surgery, we were able to observe that the robotic approach seems to be associated with a lower number of conversions due to abdominal adhesions compared to laparoscopic surgery, with an overall OR of 1.5.

However, to reduce the heterogeneity in the included studies, we performed subgroups analyses to assess if the statistical significance was confirmed in each surgical field.

Our subgroups analysis performed on colorectal patients confirmed the reduced conversion rate due to adhesions in the robotic surgery population, as obtained in the overall analysis, with an OR of 2.22 (95% CI 1.18–4.19, *p* = 0.01). Furthermore, the analysis on different colorectal procedures showed that this significance was present only in colorectal procedures performed in cancer patients (OR 2.62, 95% CI 1.20–5.72, *p* = 0.02), while the colorectal procedures for other diseases did not significantly impact the results (OR 1.72, 95% CI 0.27–11.16, *p* = 0.57 for rectal prolapse and OR 1.36, 95% CI 0.10–18.02, *p* = 0.81 for diverticular disease).

One potential explanation of these findings is that surgery for colorectal cancer often requires access to various quadrants of the abdomen: frequently both the supra- and the infra-mesocolic spaces. Thus, the presence of adhesions in those cases could significantly affect this type of surgical procedure, more than other speciality procedures that are confined to one compartment in the abdomen or the pelvis.

Evaluating the role of surgeons’ experience was of paramount importance, being a potential confounding factor considering the study’s primary endpoint (conversion to open). We performed this subgroup analysis to ensure that the results of the two techniques were comparable and not affected by different experience levels.

Our results showed that the robotic approach significantly reduced the conversion rate in the case of expert surgeons (OR 1.48, 95% CI 1.03–2.12, *p* = 0.03), while no significant difference was found in the case of procedures performed by “novice” surgeons (OR 1.53, 95% CI 0.44–5.28, *p* = 0.50). This finding was also observed in the overall conversion analysis and in the colorectal surgery subgroup.

In the analysis on the colorectal surgery subgroup performed by expert surgeons, a statistically significant difference favouring robotic surgery was observed (OR 2.34, 95% CI 1.07–5.11, *p* = 0.03), while no statistically significant difference was observed among colorectal (OR 1.35, 95% CI 0.25–7.40, *p* = 0.73) “novice” surgeons. One possible explanation of these results is that the benefits of the robotic approach in colorectal surgery are maximised and become evident only after completing the learning curve.

However, the criteria to define the expertise remains heterogeneous. In fact, only five studies reported the number of procedures [45,46,61,65,84] performed by the surgeons, and none of the studies provided an exact definition of the various steps of the surgical procedure. We attempted to apply rigorous criteria to evaluate the quality of the techniques, but none of the studies reported on surgeons’ credentialing, standardisation of techniques and objective evaluation and monitoring of surgeons’ skills. Nevertheless, the pooled data in this study highlight the importance of optimal training in robotic surgery in order to achieve the maximum benefits for the patients.

Our study has several strengths. To date, this is the first meta-analysis on the risk of conversion due to intraabdominal adhesions comparing robotic and laparoscopic surgery. In this setting, clinical decisions of adopting one technique over the other could be supported by our meta-analysis, which comprises a large number of studies and cases and therefore enhances the external validity and generalizability.

Based on these results, we could encourage the use of robotic surgery in patients with known or suspected abdominal adhesions and due to undergo a colorectal cancer resection.

However, several limitations should also be acknowledged. By only including studies published in English with full text, a language bias could not be excluded. Results from retrospective studies inevitably contained potential selection bias, confounding bias and missing data bias.

We could not fully adjust for confounding factors, including the causes and the extent and severity of the adhesions. Additionally, in the included studies, the definition of expertise is heterogeneous, with an increased risk of surgeons-dependent performance bias.

Further efforts are required to implement a quality assurance framework when reporting on advanced surgical skills [21].

No ad hoc studies were currently available specifically addressing the role of robotic versus laparoscopic surgery determining conversion related to adhesions.

Additionally, the definition of conversion to open was not adequately standardised; in fact, only nine studies provide this information. [13,35,41,43,45,46,50,78,84] and an optimal information prevalence of conversions for adhesions cannot be obtained.

## 5. Conclusions

Limitations notwithstanding, this state-of-the-art review provides a lens through which to scrutinise and appraise the currently available evidence on abdominal robotic and laparoscopic surgery with a focus on conversion rates due to intraabdominal adhesions.

Our study should not be interpreted as an arbitrary conclusion that any planned colorectal intervention with certain or presumed adhesions should be treated by a robotic approach. Instead, our findings should support surgeons in the process of selecting the optimal technique and highlight the potential advantages of the robotic approach when performing surgery with a high risk of necessitating complex adhesiolysis. 

## Figures and Tables

**Table 1:** NOS quality assessment of the included non-randomised trials.

Study	Selection				Comparability	Outcome			Total
	Representativeness of Exposed Cohort	Selection of the Non-Exposed Cohort	Ascertainment of Exposure	Outcome Not Present at the Start of the Study		Assessment of Outcome	Length of Follow-Up	Adequacy of Follow-Up	
Albassam A.A. et al., 2009 [25]		*	*	*	*	*	*	*	*******
Alfieri S. et al., 2019 [26]	*	*	*	*	**	*	*	*	*********
Alhossaini R.M. et al., 2019 [27]	*	*	*	*	*	*	*	*	********
Alimi Q. et al., 2018 [28]		*	*	*	*	*	*	*	*******
Ayloo S. et al., 2011 [29]		*	*	*	*	*	*	*	*******
Beak J. et al., 2010 [30]	*	*	*	*	**	*	*	*	*********
Benizri E.I. et al., 2013 [31]		*	*	*	*	*	*	*	*******
Benway B.M. et al., 2009 [32]		*	*	*	*	*	*	*	*******
Bilgin I.A. et al., 2019 [33]		*	*	*	**	*	*	*	********
Boggess J.F. et al., 2008 [34]		*	*	*	*	*	*	*	*******
Buchs N.C. et al., 2014 [35]		*	*	*	**	*	*	*	********
Butturini G. et al., 2014 [36]	*	*	*	*	**	*	*	*	*********
Cassini D. et al., 2018 [37]	*	*	*	*	**	*	*	*	*********
Chiu L.H. et al., 2015 [38]	*	*	*	*	*	*	*	*	********
Coronado P.J. et al., 2012 [39]		*	*	*	**	*	*	*	********
Corrado G. et al., 2018 [40]		*	*	*	*	*	*	*	*******
Crippa J. et al., 2019 [41]		*	*	*	**	*	*	*	********
Cuendis-Velazquez A. et al., 2018 [42]	*	*	*	*	*	*	*	*	********
Elliott P.A. et al., 2015 [43]	*	*	*	*	**	*	*	*	*********
Escobar F. et al., 2011 [44]		*	*	*	*	*	*	*	*******
Esen E. et al., 2018 [45]	*	*	*	*	**	*	*	*	*********
Feroci F. et al., 2016 [46]		*	*	*	**	*	*	*	********
Gallotta V. et al., 2018 [47]	*	*	*	*	*	*	*	*	********
Gangemi A. et al., 2017 [48]		*	*	*	**	*	*	*	********
Gao Y. et al., 2018 [49]	*	*	*	*	**	*	*	*	*********
Goçmen A. et al., 2012 [50]	*	*	*	*	*	*	*	*	********
Goh B.K.P. et al., 2016 [13]	*	*	*	*	**	*	*	*	*********
Golcoechea J.C. et al., 2010 [51]	*	*	*	*	*	*	*	*	********
Gorgun E. et al., 2016 [52]	*	*	*	*	**	*	*	*	*********
Gray K.D. et al., 2018 [53]	*	*	*	*	**	*	*	*	*********
Guillotrean et al., 2012 [54]		*	*	*	**	*	*	*	********
Hoekstra A.V. et al., 2009 [55]		*	*	*	**	*	*	*	********
Holz D.O. et al., 2010 [56]		*	*	*	*	*	*	*	*******
Ielpo B. et al., 2014 [57]		*	*	*	**	*	*	*	********
Johnson L. et al., 2016 [58]	*	*	*	*	**	*	*	*	*********
Karabulut K.K. et al., 2012 [59]	*	*	*	*	*	*	*	*	********
Kilic G. et al., 2011 [60]	*	*	*	*	*	*	*	*	********
Kim J.C. et al., 2018 [61]	*	*	*	*	**	*	*	*	*********
Kim Y.W. et al., 2015 [62]	*	*	*	*	*	*	*	*	********
Kong Y. et al., 2019 [63]	*	*	*	*	**	*	*	*	*********
Krucharoen U. et al., 2019 [64]		*	*	*	**	*	*	*	********
Law W.L. et al., 2016 [65]	*	*	*	*	**	*	*	*	*********
Lee S.Y. et al., 2014 [66]		*	*	*	**	*	*	*	********
Leitao M.M. et al., 2012 [67]		*	*	*	*	*	*	*	*******
Lim P.C. et al., 2010 [68]		*	*	*	*	*	*	*	*******
Liu et al., 2016 [69]		*	*	*	*	*	*	*	*******
Maenpaa M.M. et al., 2016 [70]	*	*	*	*	*	*	*	*	********
Mantoo S. et al., 2013 [71]		*	*	*	**	*	*	*	********
Mehmood R.K. et al., 2014 [72]	*	*	*	*	*	*	*	*	********
Montalti R. et al., 2014 [73]		*	*	*	**	*	*	*	********
Morelli L. et al., 2016 [74]		*	*	*	*	*	*	*	*******
Najafi N. et al., 2020 [75]	*	*	*	*	**	*	*	*	*********
Nezhat F.R. et al., 2014 [76]	*	*	*	*	*	*	*	*	********
Niglio A. et al., 2019 [77]		*	*	*	**	*	*	*	********
Ozben V. et al., 2019 [78]	*	*	*	*	**	*	*	*	*********
Park J.Y. et al., 2015 [79]		*	*	*	*	*	*	*	*******
Ramji K.M. et al., 2015 [80]	*	*	*	*	*	*	*	*	********
Rencuzogullari A. et al., 2016 [81]		*	*	*	**	*	*	*	********
Seror J. et al., 2016 [82]	*	*	*	*	**	*	*	*	*********
Smith A.L. 2012 [83]	*	*	*	*	*	*	*	*	********
Spinoglio G. et al., 2018 [84]	*	*	*	*	**	*	*	*	*********
Troisi R.I. et al., 2013 [85]	*	*	*	*		*	*	*	*******
Turunen H. et al., 2013 [86]		*	*	*	**	*	*	*	********
Vasilescu C. et al., 2012 [87]		*	*	*	*	*	*	*	*******
Wang A.J. et al., 2009 [88]		*	*	*	**	*	*	*	********
Wang Z.Z. et al., 2019 [89]	*	*	*	*	**	*	*	*	*********
Warren J.A. et al., 2016 [90]	*	*	*	*	**	*	*	*	*********
Wong M.T.C. et al., 2011 [91]		*	*	*	*	*	*	*	*******
Yamaguchi T. et al., 2015 [92]	*	*	*	*	**	*	*	*	*********
Zhao X. et al., 2018 [93]		*	*	*	*	*	*	*	*******

**Table 2:** Characteristics of the included studies.

Study	Design	Patients		Surgical Field	Pathology	Procedure	Expertise
		Lap	Rob				
Albassam A.A., 2009 [25]	Retrospective	25	25	Oesophago-gastric	GERD	Nissen fundoplication	Expert
Alfieri S. et al., 2019 [26]	Retrospective	85	96	Pancreatic	pNETs	Distal pancreatectomy	Expert
Alhossaini R.M. et al., 2019 [27]	Retrospective	30	25	Oesophago-gastric	Remnant gastric cancer	Completion total gastrectomy	NR
Alimi Q. et al., 2018 [28]	Prospective	50	50	Urologic	Renal tumour	Partial nephrectomy	Expert
Ayloo S. et al., 2011 [29]	Prospective	39	30	Oesophago-gastric	Morbid obesity	Sleeve gastrectomy	NR
Beak J. et al., 2010 [30]	Prospective	41	41	Colorectal	Rectal cancer	Rectal resection with TME	Early
Benizri E.I. et al., 2013 [31]	Prospective	100	100	Oesophago-gastric	Morbid obesity	Roux-en-Y gastric bypass	Expert
Benway B.M. [32]	Retrospective	118	129	Urologic	Renal tumour	Partial nephrectomy	Expert
Bilgin I.A. et al., 2019 [33]	Retrospective	22	20	Colorectal	Diverticular disease	Sigmoidectomy	Expert
Boggess J.F. et al., 2008 [34]	Prospective	81	103	Gynaecologic	Endometrial cancer	Hysterectomy	Early
Buchs N.C. et al., 2014 [35]	Prospective	389	388	Oesophago-gastric	Morbid obesity	Roux-en-Y gastric bypass	Expert
Butturini G. et al., 2014 [36]	Prospective	21	22	Pancreatic	Pancreatic tumours	Distal pancreatectomy	Expert
Cassini D. et al., 2018 [37]	Retrospective	92	64	Colorectal	Diverticular disease	Sigmoidectomy	Expert
Chiu L.H. et al., 2015 [38]	Prospective	128	88	Gynaecologic	Benign pathology or carcinoma IS	Hysterectomy	NR
Coronado P.J. et al., 2012 [39]	Retrospective	84	71	Gynaecologic	Endometrial cancer	Hysterectomy with bilateral salpingo-oophorectomy	NR
Corrado G. et al., 2018 [40]	Retrospective	406	249	Gynaecologic	Low-grade endometrial carcinoma	Hysterectomy	Expert
Crippa J. et al., 2019 [41]	Retrospective	283	317	Colorectal	Rectal cancer	LAR or APR with TME	Expert
Cuendis-Velazquez A. et al., 2018 [42]	Retrospective	40	35	Hepatobiliary	Bile duct injury	Hepaticojejunostomy	NR
Elliott P.A. et al., 2015 [43]	Retrospective	20	11	Colorectal	Diverticulitis	Sigmoidectomy	Expert
Escobar P.F. et al., 2011 [44]	Retrospective	30	30	Gynaecologic	Endometrial cancer	Hysterectomy	Expert
Esen E. et al., 2018 [45]	Retrospective	78	100	Colorectal	Rectal cancer	Rectal resection with TME	Expert
Feroci F. et al., 2016 [46]	Retrospective	58	53	Colorectal	Rectal cancer	Rectal resection with TME	Expert
Gallotta V. et al., 2018 [47]	Retrospective	140	70	Gynaecologic	Early cervical cancer	Hysterectomy	Expert
Gangemi A. et al., 2017 [48]	Retrospective	289	676	Hepatobiliary	Cholelithiasis/cholecystitis	Cholecystectomy	Expert
Gao Y. et al., 2018 [49]	Retrospective	163	163	Oesophago-gastric	Gastric cancer	Partial and total gastrectomy	Expert
Goh B.K.P. et al., 2016 [13]	Retrospective	31	8	Pancreatic	Pancreatic tumours	Distal pancreatectomy	Early
Goioechea J.C. et al., 2010 [51]	Retrospective	173	102	Gynaecologic	Endometrial cancer	Hysterectomy	Expert
Gorgun E. et al., 2016 [52]	Retrospective	27	29	Colorectal	Rectal cancer in obese patients	LAR and APR	NR
Goçmen A. et al., 2012 [50]	Prospective	60	60	Gynaecologic	Benign gynaecologic disease	Hysterectomy	NR
Gray K.D. et al., 2018 [53]	Retrospective	66	18	Oesophago-gastric	Revision of bariatric surgery	AGB, VSG, RYGB, VBG	Expert
Guillotrean J. et al., 2012 [54]	Prospective	226	210	Urologic	Small renal mass	Partial nephrectomy	NR
Hoekstra A.V. et al., 2009 [55]	Prospective	7	32	Gynaecologic	Endometrial cancer	Hysterectomy with bilateral salpingo-oophorectomy	Expert
Holtz D.O. et al., 2019 [56]	Retrospective	20	13	Gynaecologic	Endometrial cancer	Hysterectomy with bilateral salpingo-oophorectomy	Expert
Ielpo B. et al., 2017 [57]	Retrospective	112	86	Colorectal	Rectal cancer	Rectal resection	Expert
Johnson L. et al., 2016 [58]	Retrospective	187	353	Gynaecologic	Endometrial cancer	Hysterectomy	NR
Karabulut K.K. et al., 2012 [59]	Prospective	50	50	Endocrine	Pheochromocytoma	Adrenalectomy	Expert
Kilic G.S. et al., 2011 [60]	Prospective	34	25	Gynaecologic	Benign gynaecologic disease	Hysterectomy	Expert
Kim J.C. et al., 2018 [61]	Prospective	53	20	Colorectal	Colon cancer	Left colectomy	NR
Kim Y.W. et al., 2015 [62]	Prospective	288	87	Oesophago-gastric	Gastric cancer	Distal gastrectomy	Expert
Kong Y. et al., 2019 [63]	Retrospective	532	266	Oesophago-gastric	Gastric cancer	Partial and total gastrectomy	Expert
Krucharoen U. et al., 2019 [64]	Retrospective	16	18	Vascular	Median arcuate ligament syndrome	MAL release	Expert
Law W.L. et al., 2016 [65]	Prospective	171	220	Colorectal	Rectal cancer	Hartmann procedure, LAR and APR	NR
Lee S.Y. et al., 2014 [66]	Retrospective	131	37	Pancreatic	Pancreatic tumours	Distal pancreatectomy	Expert
Leitao M.M. et al., 2012 [67]	Retrospective	302	347	Gynaecologic	Uterine cancer	Hysterectomy	Expert
Lim P.C. et al., 2019 [68]	Retrospective	122	122	Gynaecologic	Endometrial cancer	Hysterectomy	Expert
Liu et al., 2016 [69]	Retrospective	25	27	Pancreatic	Periampullary neoplasms	PD	Expert
Maenpaa M.M. et al., 2016 [70]	Rct	48	51	Gynaecologic	Low-grade endometrial carcinoma	Hysterectomy	Expert
Mantoo S. et al., 2013 [71]	Retrospective	74	44	Colorectal	Obstructed defecation	Ventral mesh rectopexy	NR
Mehmood R.K. et al., 2014 [72]	Prospective	34	17	Colorectal	Rectal prolapse	Ventral mesh rectopexy	NR
Montalti R. et al., 2015 [73]	Retrospective	72	36	Hepatobiliary	Liver diseases	Posterosuperior segments resection	Expert
Morelli L. et al., 2016 [74]	Retrospective	41	41	Endocrine	Benign or malignant adrenal tumour	Adrenalectomy	Expert
Najafi N. et al., 2020 [75]	Retrospective	40	35	Pancreatic	Benign and borderline tumours	Distal pancreatic resection and enucleation	NR
Nezhat F.R. et al., 2014 [76]	Retrospective	13	9	Gynaecologic	Early ovarian cancer	Salpingo-oophorectomy	NR
Niglio A. et al., 2019 [77]	Retrospective	64	40	Endocrine	Adrenal cancer	Adrenalectomy	NR
Ozben V. et al., 2019 [78]	Retrospective	56	26	Colorectal	Benign or malignant pathology	Subtotal or total colectomy	Expert
Park J.Y. et al., 2015 [79]	Retrospective	622	148	Oesophago-gastric	Early gastric cancer	Partial and total gastrectomy	Expert
Ramji K.M. et al., 2015 [80]	Retrospective	27	26	Colorectal	Rectal cancer	Rectal resection	Early
Rencuzogullari A. et al., 2016 [81]	Retrospective	21	21	Colorectal	IBD	Proctectomy	Early
Seror J et al., 2013 [82]	Retrospective	106	40	Gynaecologic	Endometrial cancer	Hysterectomy with bilateral salpingo-oophorectomy	Expert
Smith A.L. et al., 2012 [83]	Retrospective	106	116	Gynaecologic	Endometrial cancer	Hysterectomy	Early
Spinoglio G. et al., 2018 [84]	Retrospective	100	100	Colorectal	Right colon cancer	Right colectomy with CME	Expert
Troisi R.I. et al., 2013 [85]	Retrospective	223	40	Hepatobiliary	Liver diseases	Liver resection	Expert
Turunen H. et al., 2013 [86]	Retrospective	150	67	Gynaecologic	Endometrial cancer	Hysterectomy	Expert
Vasilescu C. et al., 2012 [87]	Retrospective	22	10	Splenic	Hereditary spherocytosis	Splenectomy	NR
Wang A.J. et al., 2009 [88]	Retrospective	62	40	Urologic	Renal cell carcinoma	Partial nephrectomy	Expert
Wang Z.Z. et al., 2019 [89]	Retrospective	48	92	Hepatobiliary	Benign or malignant hepatic lesions	Hemiepatectomy	Expert
Warren J.A. et al., 2016 [90]	Retrospective	103	53	Abdominal wall	Ventral hernia	Ventral hernia repair	NR
Wong M.T.C. et al., 2011 [91]	Prospective	40	23	Colorectal	Complex rectocele	Ventral mesh rectopexy	Expert
Yamaguchi T. et al., 2015 [92]	Retrospective	239	203	Colorectal	Rectal cancer	Rectal resection	Expert
Zhao X. et al., 2018 [93]	Retrospective	101	101	Urologic	Renal tumour	Simple enucleation with single layer renorrhaphy	Expert

GERD—gastroesophageal reflux disease; pNET—pancreatic neuroendocrine tumour; IS—in situ; IBD—intestinal bowel disease; TME—total mesorectal excision; LAR—low anterior resection; APR—abdominoperineal resection; AGB—adjustable gastric banding; VSG—vertical sleeve gastrectomy; RYGB—Roux-en-Y gastric bypass; VBG—vertical banded gastroplasty; MAL—median arcuate ligament; PD—pancreaticoduodenectomy; CME—complete mesocolic excision; NR—not reported.

**Table 3:** *p*-values of the meta-regression analysis.

Covariates	*p* Value
Mean age	0.67
Female gender	0.5
BMI	0.99
ASA Score I	0.44
ASA Score II	0.92
Tumour stage II	0.36
Tumour stage IV	0.22
Previous abdominal surgery	0.03

BMI—body mass index.

## Data Availability

The data presented in this study are available on request from the corresponding author.

## Supplementary Materials

The following supporting information can be downloaded at: https://www.mdpi.com/article/10.3390/jpm12020307/s1. Figure S1: Forest plot analysis of the included studies.

## References

[1] Milone M., Manigrasso M., Vertaldi S., Velotti N., Aprea G., Maione F., Gennarelli N., De Simone G., De Conno B., Pesce M. (2019). Robotic versus laparoscopic approach to treat symptomatic achalasia: Systematic review with meta-analysis. Dis. Esophagus.

[2] Chen K., Pan Y., Zhang B., Maher H., Wang X.F., Cai X.J. (2017). Robotic versus laparoscopic Gastrectomy for gastric cancer: A systematic review and updated meta-analysis. BMC Surg..

[3] Gavriilidis P., Wheeler J., Spinelli A., de’Angelis N., Simopoulos C., Di Saverio S. (2020). Robotic vs laparoscopic total mesorectal excision for rectal cancers: Has a paradigm change occurred? A systematic review by updated meta-analysis. Colorectal Dis..

[4] Solaini L., Bazzocchi F., Cavaliere D., Avanzolini A., Cucchetti A., Ercolani G. (2017). Robotic versus laparoscopic right colectomy: An updated systematic review and meta-analysis. Surg. Endosc..

[5] Milone M., Manigrasso M., Velotti N., Torino S., Vozza A., Sarnelli G., Aprea G., Maione F., Gennarelli N., Musella M. (2019). Completeness of total mesorectum excision of laparoscopic versus robotic surgery: A review with a meta-analysis. Int. J. Colorectal Dis..

[6] Ng A.T., Tam P.C. (2014). Current status of robot-assisted surgery. Hong Kong Med. J..

[7] Ceccarelli G., Andolfi E., Biancafarina A., Rocca A., Amato M., Milone M., Scricciolo M., Frezza B., Miranda E., De Prizio M. (2017). Robot-assisted surgery in elderly and very elderly population: Our experience in oncologic and general surgery with literature review. Aging Clin. Exp. Res..

[8] Park D.A., Lee D.H., Kim S.W., Lee S.H. (2016). Comparative safety and effectiveness of robot-assisted laparoscopic hysterectomy versus conventional laparoscopy and laparotomy for endometrial cancer: A systematic review and meta-analysis. Eur. J. Surg. Oncol..

[9] Huang Y.J., Kang Y.N., Huang Y.M., Wu A.T., Wang W., Wei P.L. (2019). Effects of laparoscopic vs robotic-assisted mesorectal excision for rectal cancer: An update systematic review and meta-analysis of randomized controlled trials. Asian J. Surg..

[10] Nota C.L., Rinkes I.H.B., Molenaar I.Q., van Santvoort H.C., Fong Y., Hagendoorn J. (2016). Robot-assisted laparoscopic liver resection: A systematic review and pooled analysis of minor and major hepatectomies. HPB.

[11] Albright B.B., Witte T., Tofte A.N., Chou J., Black J.D., Desai V.B., Erekson E.A. (2016). Robotic versus laparoscopic hysterectomy for benign disease: A systematic review and meta-analysis of randomized trials. J. Minim. Invasive Gynecol..

[12] Ind T., Laios A., Hacking M., Nobbenhuis M. (2017). A comparison of operative outcomes between standard and robotic laparoscopic surgery for endometrial cancer: A systematic review and meta-analysis. Int. J. Med Robot. Comput. Assist. Surg..

[13] Goh B.K., Chan C.Y., Soh H.L., Lee S.Y., Cheow P.C., Chow P.K., Ooi L.L., Chung A.Y. (2017). A comparison between robotic-assisted laparoscopic distal pancreatectomy versus laparoscopic distal pancreatectomy. Int. J. Med. Robot..

[14] Qu L., Zhiming Z., Xianglong T., Yuanxing G., Yong X., Rong L., Yee L.W. (2018). Short- and mid-term outcomes of robotic versus laparoscopic distal pancreatosplenectomy for pancreatic ductal adenocarcinoma: A retrospective propensity score-matched study. Int. J. Surg..

[15] Jayne D., Pigazzi A., Marshall H., Croft J., Corrigan N., Copeland J., Quirke P., West N., Rautio T., Thomassen N. (2017). Effect of robotic-assisted vs conventional laparoscopic surgery on risk of conversion to open laparotomy among patients undergoing resection for rectal cancer: The ROLARR randomized clinical trial. JAMA.

[16] Bhama A.R., Wafa A.M., Ferraro J., Collins S., Mullard A.J., Vandewarker J.F., Krapohl G., Byrn J.C., Cleary R.K. (2016). Comparison of risk factors for unplanned conversion from laparoscopic and robotic to open colorectal surgery using the Michigan surgical quality collaborative (MSQC) database. J. Gastrointest. Surg..

[17] Jones N., Fleming N.D., Nick A.M., Munsell M.F., Rallapalli V., Westin S.N., Meyer L.A., Schmeler K.M., Ramirez P.T., Soliman P.T. (2014). Conversion from robotic surgery to laparotomy: A case-control study evaluating risk factors for conversion. Gynecol. Oncol..

[18] Unger C.A., Lachiewicz M.P., Ridgeway B. (2016). Risk factors for robotic gynecologic procedures requiring conversion to other surgical procedures. Int. J. Gynaecol. Obstet..

[19] Page M.J., McKenzie J.E., Bossuyt P.M., Boutron I., Hoffmann T.C., Mulrow C.D., Shamseer L., Tetzlaff J.M., Akl E.A., Brennan S.E. (2021). The PRISMA 2020 statement: An updated guideline for reporting systematic reviews. BMJ.

[20] Stroup D.F., Berlin J.A., Morton S.C., Olkin I., Williamson G.D., Rennie D., Moher D., Becker B.J., Sipe T.A., Thacker S.B. (2000). Meta-analysis of observational studies in epidemiology: A proposal for reporting. Meta-analysis of observational studies in epidemiology (MOOSE) group. JAMA.

[21] Francis N.K., Curtis N.J., Crilly L., Noble E., Dyke T., Hipkiss R., Dalton R., Allison A., Salib E., Ockrim J. (2018). Does the number of operating specialists influence the conversion rate and outcomes after laparoscopic colorectal cancer surgery?. Surg. Endosc..

[22] Foster J.D., Mackenzie H., Nelson H., Hanna G.B., Francis N.K. (2014). Methods of quality assurance in multicenter trials in laparoscopic colorectal surgery: A systematic review. Ann. Surg..

[23] Wells G.A., Shea B., O’Connell D., Peterson J., Welch V., Losos M., Tugwell P., Ottawa Hospital Research Institute The Newcastle-Ottawa Scale (NOS) for Assessing the Quality of Nonrandomized Studies in Meta-Analyses. http://www.ohri.ca/programs/clinical_epidemiology/oxford.html.

[24] Higgins J.P., Altman D.G., Gøtzsche P.C., Jüni P., Moher D., Oxman A.D., Savović J., Schulz K.F., Weeks L., Sterne J.A. (2011). The Cochrane collaboration’s tool for assessing risk of bias in randomised trials. BMJ.

[25] Albassam A.A., Mallick M.S., Gado A., Shoukry M. (2009). Nissen fundoplication, robotic-assisted versus laparoscopic procedure: A comparative study in children. Eur. J. Pediatr. Surg..

[26] Alfieri S., Butturini G., Boggi U., Pietrabissa A., Morelli L., Vistoli F., Damoli I., Peri A., Fiorillo C., The Italian Robotic pNET Group (2019). Short-term and long-term outcomes after robot-assisted versus laparoscopic distal pancreatectomy for pancreatic neuroendocrine tumors (pNETs): A multicenter comparative study. Langenbeck’s Arch. Surg..

[27] Alhossaini R.M., Altamran A.A., Cho M., Roh C., Seo W.J., Choi S., Son T., Kim H.-I., Hyung W.J. (2020). Lower rate of conversion using robotic-assisted surgery compared to laparoscopy in completion total gastrectomy for remnant gastric cancer. Surg. Endosc..

[28] Alimi Q., Peyronnet B., Sebe P., Cote J.-F., Kammerer-Jacquet S.-F., Khene Z.-E., Pradere B., Mathieu R., Verhoest G., Guillonneau B. (2018). Comparison of short-term functional, oncological, and perioperative outcomes between laparoscopic and robotic partial nephrectomy beyond the learning curve. J. Laparoendosc. Adv. Surg. Tech..

[29] Ayloo S., Buchs N.C., Addeo P., Bianco F.M., Giulianotti P.C. (2011). Robot-assisted sleeve gastrectomy for super-morbidly obese patients. J. Laparoendosc. Adv. Surg. Tech..

[30] Baek J.H., Pastor C., Pigazzi A. (2011). Robotic and laparoscopic total mesorectal excision for rectal cancer: A case-matched study. Surg. Endosc..

[31] Benizri E.I., Renaud M., Reibel N., Germain A., Ziegler O., Zarnegar R., Ayav A., Bresler L., Brunaud L. (2013). Perioperative outcomes after totally robotic gastric bypass: A prospective nonrandomized controlled study. Am. J. Surg..

[32] Benway B.M., Bhayani S.B., Rogers C.G., Dulabon L.M., Patel M.N., Lipkin M., Wang A.J., Stifelman M.D. (2009). Robot assisted partial nephrectomy versus laparoscopic partial nephrectomy for renal tumors: A multi-institutional analysis of perioperative outcomes. J. Urol..

[33] Bilgin I.A., Bas M., Benlice C., Esen E., Ozben V., Aytac E., Baca B., Hamzaoglu I., Karahasanoglu T. (2020). Totally laparoscopic and totally robotic surgery in patients with left-sided colonic diverticulitis. Int. J. Med. Robot..

[34] Boggess J.F., Gehrig P.A., Cantrell L., Shafer A., Ridgway M., Skinner E.N., Fowler W.C. (2008). A comparative study of 3 surgical methods for hysterectomy with staging for endometrial cancer: Robotic assistance, laparoscopy, laparotomy. Am. J. Obstet. Gynecol..

[35] Buchs N.C., Morel P., Azagury D., Jung M.K., Chassot G., Huber O., Hagen M.E., Pugin F.L. (2014). Laparoscopic versus robotic Roux-en-Y gastric bypass: Lessons and long-term follow-up learned from a large prospective monocentric study. Obes. Surg..

[36] Butturini G., Damoli I., Crepaz L., Malleo G., Marchegiani G., Daskalaki D., Esposito A., Cingarlini S., Salvia R., Bassi C. (2015). A prospective non-randomised single-center study comparing laparoscopic and robotic distal pancreatectomy. Surg. Endosc..

[37] Cassini D., Depalma N., Grieco M., Cirocchi R., Manoochehri F., Baldazzi G. (2019). Robotic pelvic dissection as surgical treatment of complicated diverticulitis in elective settings: A comparative study with fully laparoscopic procedure. Surg. Endosc..

[38] Chiu L.H., Chen C.H., Tu P.C., Chang C.W., Yen Y.K., Liu W.M. (2015). Comparison of robotic surgery and laparoscopy to perform total hysterectomy with pelvic adhesions or large uterus. J. Minim. Access Surg..

[39] Coronado P.J., Herraiz M.A., Magrina J.F., Fasero M., Vidart J.A. (2012). Comparison of perioperative outcomes and cost of robotic-assisted laparoscopy, laparoscopy and laparotomy for endometrial cancer. Eur. J. Obstet. Gynecol. Reprod. Biol..

[40] Corrado G., Vizza E., Cela V., Mereu L., Bogliolo S., Legge F., Ciccarone F., Mancini E., Gallotta V., Baiocco E. (2018). Laparoscopic versus robotic hysterectomy in obese and extremely obese patients with endometrial cancer: A multi-institutional analysis. Eur. J. Surg. Oncol..

[41] Crippa J., Grass F., Achilli P., Mathis K.L., Kelley S.R., Merchea A., Colibaseanu D.T., Larson D.W. (2020). Risk factors for conversion in laparoscopic and robotic rectal cancer surgery. Br. J. Surg..

[42] Cuendis-Velázquez A., Trejo-Ávila M., Bada-Yllán O., Cárdenas-Lailson E., Morales-Chávez C., Fernández-Álvarez L., Romero-Loera S., Rojano-Rodríguez M., Valenzuela-Salazar C., Moreno-Portillo M. (2019). A new era of bile duct repair: Robotic-assisted versus laparoscopic hepaticojejunostomy. J. Gastrointest. Surg..

[43] Elliott P.A., McLemore E.C., Abbass M.A., Abbas M.A. (2015). Robotic versus laparoscopic resection for sigmoid diverticulitis with fistula. J. Robot. Surg..

[44] Escobar P.F., Frumovitz M., Soliman P.T., Frasure H.E., Fader A.N., Schmeler K.M., Ramirez P.T. (2012). Comparison of single-port laparoscopy, standard laparoscopy, and robotic surgery in patients with endometrial cancer. Ann. Surg. Oncol..

[45] Esen E., Aytac E., Ağcaoğlu O., Zenger S., Balik E., Baca B., Hamzaoğlu I., Karahasanoğlu T., Buğra D. (2018). Totally robotic versus totally laparoscopic surgery for rectal cancer. Surg. Laparosc. Endosc. Percutaneous Tech..

[46] Feroci F., Vannucchi A., Bianchi P.P., Cantafio S., Garzi A., Formisano G., Scatizzi M. (2016). Total mesorectal excision for mid and low rectal cancer: Laparoscopic vs robotic surgery. World J. Gastroenterol..

[47] Gallotta V., Conte C., Federico A., Vizzielli G., Alletti S.G., Tortorella L., Anchora L.P., Cosentino F., Chiantera V., Fagotti A. (2018). Robotic versus laparoscopic radical hysterectomy in early cervical cancer: A case matched control study. Eur. J. Surg. Oncol..

[48] Gangemi A., Danilkowicz R., Elli F.E., Bianco F., Masrur M., Giulianotti P.C. (2017). Could ICG-aided robotic cholecystectomy reduce the rate of open conversion reported with laparoscopic approach? A head to head comparison of the largest single institution studies. J. Robot. Surg..

[49] Gao Y., Xi H., Qiao Z., Li J., Zhang K., Xie T., Shen W., Cui J., Wei B., Chen L. (2019). Comparison of robotic- and laparoscopic-assisted gastrectomy in advanced gastric cancer: Updated short- and long-term results. Surg. Endosc..

[50] Göçmen A., Şanlıkan F., Uçar M.G. (2012). Robot-assisted hysterectomy vs total laparoscopic hysterectomy: A comparison of short-term surgical outcomes. Int. J. Med. Robot..

[51] Cardenas-Goicoechea J., Adams S., Bhat S.B., Randall T.C. (2010). Surgical outcomes of robotic-assisted surgical staging for endometrial cancer are equivalent to traditional laparoscopic staging at a minimally invasive surgical center. Gynecol. Oncol..

[52] Gorgun E., Ozben V., Costedio M., Stocchi L., Kalady M., Remzi F. (2016). Robotic versus conventional laparoscopic rectal cancer surgery in obese patients. Colorectal Dis..

[53] Gray K.D., Moore M.D., Elmously A., Bellorin O., Zarnegar R., Dakin G., Pomp A., Afaneh C. (2018). Perioperative outcomes of laparoscopic and robotic revisional bariatric surgery in a complex patient population. Obes. Surg..

[54] Guillotreau J., Haber G.-P., Autorino R., Miocinovic R., Hillyer S., Hernandez A., Laydner H., Yakoubi R., Isac W., Long J.-A. (2012). Robotic partial nephrectomy versus laparoscopic cryoablation for the small renal mass. Eur. Urol..

[55] Hoekstra A.V., Jairam-Thodla A., Rademaker A., Singh D.K., Buttin B.M., Lurain J.R., Schink J.C., Lowe M.P. (2009). The impact of robotics on practice management of endometrial cancer: Transitioning from traditional surgery. Int. J. Med. Robot..

[56] Holtz D.O., Miroshnichenko G., Finnegan M.O., Chernick M., Dunton C.J. (2010). Endometrial cancer surgery costs: Robot vs laparoscopy. J. Minim. Invasive Gynecol..

[57] Ielpo B., Duran H., Diaz E., Fabra I., Caruso R., Malavé L., Ferri V., Nuñez J., Ruiz-Ocaña A., Jorge E. (2017). Robotic versus laparoscopic surgery for rectal cancer: A comparative study of clinical outcomes and costs. Int. J. Colorectal Dis..

[58] Johnson L., Bunn W.D., Nguyen L., Rice J., Raj M., Cunningham M.J. (2017). Clinical comparison of robotic, laparoscopic, and open hysterectomy procedures for endometrial cancer patients. J. Robot. Surg..

[59] Karabulut K., Agcaoglu O., Aliyev S., Siperstein A., Berber E. (2012). Comparison of intraoperative time use and perioperative outcomes for robotic versus laparoscopic adrenalectomy. Surgery.

[60] Kilic G.S., Moore G., Elbatanony A., Radecki C., Phelps J.Y., Borahay M.A. (2011). Comparison of perioperative outcomes of total laparoscopic and robotically assisted hysterectomy for benign pathology during introduction of a robotic program. Obstet. Gynecol. Int..

[61] Kim J.C., Lee J.L., Yoon Y.S., Kim C.W., Park I.J., Lim S.B. (2018). Robotic left colectomy with complete mesocolectomy for splenic flexure and descending colon cancer, compared with a laparoscopic procedure. Int. J. Med. Robot..

[62] Kim Y.-W., Reim D., Park J.Y., Eom B.W., Kook M.-C., Ryu K.W., Yoon H.M. (2016). Role of robot-assisted distal gastrectomy compared to laparoscopy-assisted distal gastrectomy in suprapancreatic nodal dissection for gastric cancer. Surg. Endosc..

[63] Kong Y., Cao S., Liu X., Li Z., Wang L., Lu C., Shen S., Zhu H., Zhou Y. (2020). Short-term clinical outcomes after laparoscopic and robotic gastrectomy for gastric cancer: A propensity score matching analysis. J. Gastrointest. Surg..

[64] Khrucharoen U., Juo Y.Y., Chen Y., Jimenez J.C., Dutson E.P. (2020). Short- and intermediate-term clinical outcome comparison between laparoscopic and robotic-assisted median arcuate ligament release. J. Robot. Surg..

[65] Law W.L., Foo D.C.C. (2017). Comparison of short-term and oncologic outcomes of robotic and laparoscopic resection for mid- and distal rectal cancer. Surg. Endosc..

[66] Lee S.Y., Allen P.J., Sadot E., D’Angelica M.I., DeMatteo R.P., Fong Y., Jarnagin W.R., Kingham T.P. (2015). Distal pancreatectomy: A single institution’s experience in open, laparoscopic, and robotic approaches. J. Am. Coll. Surg..

[67] Leitao M.M., Briscoe G., Santos K., Winder A., Jewell E.L., Hoskins W.J., Chi D.S., Abu-Rustum N.R., Sonoda Y., Brown C.L. (2012). Introduction of a computer-based surgical platform in the surgical care of patients with newly diagnosed uterine cancer: Outcomes and impact on approach. Gynecol. Oncol..

[68] Lim P.C., Kang E., Park D.H. (2011). A comparative detail analysis of the learning curve and surgical outcome for robotic hysterectomy with lymphadenectomy versus laparoscopic hysterectomy with lymphadenectomy in treatment of endometrial cancer: A case-matched controlled study of the first one hundred twenty two patients. Gynecol. Oncol..

[69] Liu R., Zhang T., Zhao Z.M., Tan X.L., Zhao G.D., Zhang X., Xu Y. (2017). The surgical outcomes of robot-assisted laparoscopic pancreaticoduodenectomy versus laparoscopic pancreaticoduodenectomy for periampullary neoplasms: A comparative study of a single center. Surg. Endosc..

[70] Mäenpää M.M., Nieminen K., Tomás E.I., Laurila M., Luukkaala T.H., Mäenpää J.U. (2016). Robotic-assisted vs traditional laparoscopic surgery for endometrial cancer: A randomized controlled trial. Am. J. Obstet. Gynecol..

[71] Mantoo S., Podevin J., Regenet N., Rigaud J., Lehur P.A., Meurette G. (2013). Is robotic-assisted ventral mesh rectopexy superior to laparoscopic ventral mesh rectopexy in the management of obstructed defaecation?. Colorectal Dis..

[72] Mehmood R.K., Parker J., Bhuvimanian L., Qasem E., Mohammed A.A., Zeeshan M., Grugel K., Carter P., Ahmed S. (2014). Short-term outcome of laparoscopic versus robotic ventral mesh rectopexy for full-thickness rectal prolapse. Is robotic superior?. Int. J. Colorectal Dis..

[73] Montalti R., Scuderi V., Patriti A., Vivarelli M., Troisi R.I. (2016). Robotic versus laparoscopic resections of posterosuperior segments of the liver: A propensity score-matched comparison. Surg. Endosc..

[74] Morelli L., Tartaglia D., Bronzoni J., Palmeri M., Guadagni S., Gennai A., Bianchini M., Bastiani L., Moglia A., Fommei E. (2016). Robotic assisted versus pure laparoscopic surgery of the adrenal glands: A case-control study comparing surgical techniques. Langenbeck’s Arch. Surg..

[75] Najafi N., Mintziras I., Wiese D., Albers M.B., Maurer E., Bartsch D.K. (2020). A retrospective comparison of robotic versus laparoscopic distal resection and enucleation for potentially benign pancreatic neoplasms. Surg. Today.

[76] Nezhat F.R., Finger T.N., Vetere P., Radjabi A.R., Vega M., Averbuch L., Khalil S., Altinbas S.K., Lax D. (2014). Comparison of perioperative outcomes and complication rates between conventional versus robotic-assisted laparoscopy in the evaluation and management of early, advanced, and recurrent stage ovarian, fallopian tube, and primary peritoneal cancer. Int. J. Gynecol. Cancer.

[77] Niglio A., Grasso M., Costigliola L., Zenone P., De Palma M. (2020). Laparoscopic and robot-assisted transperitoneal lateral adrenalectomy: A large clinical series from a single center. Updates Surg..

[78] Ozben V., De Muijnck C., Karabork M., Ozoran E., Zenger S., Bilgin I.A., Aytac E., Baca B., Balik E., Hamzaoglu I. (2019). The da Vinci Xi system for robotic total/subtotal colectomy vs. conventional laparoscopy: Short-term outcomes. Tech. Coloproctol..

[79] Park J.Y., Ryu K.W., Reim D., Eom B.W., Yoon H.M., Rho J.Y., Choi I.J., Kim Y.-W. (2015). Robot-assisted gastrectomy for early gastric cancer: Is it beneficial in viscerally obese patients compared to laparoscopic gastrectomy?. World J. Surg..

[80] Ramji K.M., Cleghorn M.C., Josse J.M., MacNeill A., O’brien C., Urbach D., Quereshy F.A. (2016). Comparison of clinical and economic outcomes between robotic, laparoscopic, and open rectal cancer surgery: Early experience at a tertiary care center. Surg. Endosc..

[81] Rencuzogullari A., Gorgun E., Costedio M., Aytac E., Kessler H., Abbas M.A., Remzi F.H. (2016). Case-matched comparison of robotic versus laparoscopic proctectomy for inflammatory bowel disease. Surg. Laparosc. Endosc. Percutaneous Tech..

[82] Seror J., Bats A.S., Huchon C., Bensaïd C., Douay-Hauser N., Lécuru F. (2014). Laparoscopy vs robotics in surgical management of endometrial cancer: Comparison of intraoperative and postoperative complications. J. Minim. Invasive Gynecol..

[83] Smith A.L., Krivak T.C., Scott E.M., Rauh-Hain J.A., Sukumvanich P., Olawaiye A.B., Richard S.D. (2012). Dual-console robotic surgery compared to laparoscopic surgery with respect to surgical outcomes in a gynecologic oncology fellowship program. Gynecol. Oncol..

[84] Spinoglio G., Bianchi P.P., Marano A., Priora F., Lenti L.M., Ravazzoni F., Petz W., Borin S., Ribero D., Formisano G. (2018). Robotic versus laparoscopic right colectomy with complete mesocolic excision for the treatment of colon cancer: Perioperative outcomes and 5-year survival in a consecutive series of 202 patients. Ann. Surg. Oncol..

[85] Troisi R.I., Patriti A., Montalti R., Casciola L. (2013). Robot assistance in liver surgery: A real advantage over a fully laparoscopic approach? Results of a comparative bi-institutional analysis. Int. J. Med. Robot..

[86] Turunen H., Pakarinen P., Sjöberg J., Loukovaara M. (2013). Laparoscopic vs robotic-assisted surgery for endometrial carcinoma in a centre with long laparoscopic experience. J. Obstet. Gynaecol..

[87] Vasilescu C., Stanciulea O., Tudor S. (2012). Laparoscopic versus robotic subtotal splenectomy in hereditary spherocytosis. Potential advantages and limits of an expensive approach. Surg. Endosc..

[88] Wang A.J., Bhayani S.B. (2009). Robotic partial nephrectomy versus laparoscopic partial nephrectomy for renal cell carcinoma: Single-surgeon analysis of >100 consecutive procedures. Urology.

[89] Wang Z.Z., Tang W.B., Hu M.G., Zhao Z.M., Zhao G.D., Li C.G., Tan X.L., Zhang X., Lau W.Y., Liu R. (2019). Robotic vs laparoscopic hemihepatectomy: A comparative study from a single center. J. Surg. Oncol..

[90] Warren J.A., Cobb W.S., Ewing J.A., Carbonell A.M. (2017). Standard laparoscopic versus robotic retromuscular ventral hernia repair. Surg. Endosc..

[91] Wong M.T., Meurette G., Rigaud J., Regenet N., Lehur P.A. (2011). Robotic versus laparoscopic rectopexy for complex rectocele: A prospective comparison of short-term outcomes. Dis. Colon. Rectum..

[92] Yamaguchi T., Kinugasa Y., Shiomi A., Tomioka H., Kagawa H., Yamakawa Y. (2016). Robotic-assisted vs. conventional laparoscopic surgery for rectal cancer: Short-term outcomes at a single center. Surg. Today.

[93] Zhao X., Lu Q., Campi R., Ji C., Guo S., Liu G., Zhang S., Li X., Gan W., Minervini A. (2018). Endoscopic robot-assisted simple enucleation versus laparoscopic simple enucleation with single-layer renorrhaphy in localized renal tumors: A propensity score-matched analysis from a high-volume centre. Urology.

[94] Friedrich J.O., Adhikari N.K., Beyene J. (2007). Inclusion of zero total event trials in meta-analyses maintains analytic consistency and incorporates all available data. BMC Med. Res. Methodol..

[95] Furukawa T.A., Barbui C., Cipriani A., Brambilla P., Watanabe N. (2006). Imputing missing standard deviations in meta-analyses can provide accurate results. J. Clin. Epidemiol..

[96] DerSimonian R., Laird N. (1986). Meta-analysis in clinical trials. Control Clin. Trials.

[97] Higgins J.P., Thompson S.G., Deeks J.J., Altman D.G. (2003). Measuring inconsistency in meta-analyses. BMJ.

[98] Thompson S.G., Sharp S.J. (1999). Explaining heterogeneity in meta-analysis: A comparison of methods. Stat. Med..

[99] Egger M., Davey Smith G., Schneider M., Minder C. (1997). Bias in meta-analysis detected by a simple, graphical test. BMJ.

[100] Milone M., Manigrasso M., Burati M., Elmore U., Gennarelli N., Giglio M.C., Maione F., Musella M., Conte V.L., Milone F. (2019). Intracorporeal versus extracorporeal anastomosis after laparoscopic gastrectomy for gastric cancer. A systematic review with meta-analysis. J. Visc. Surg..

[101] Milone M., Vignali A., Milone F., Pignata G., Elmore U., Musella M., De Placido G., Mollo A., Fernandez L.M.S., Coretti G. (2015). Colorectal resection in deep pelvic endometriosis: Surgical technique and post-operative complications. World J. Gastroenterol..

[102] Milone M., Manigrasso M., Burati M., Velotti N., Milone F., De Palma G.D. (2018). Surgical resection for rectal cancer. Is laparoscopic surgery as successful as open approach? A systematic review with meta-analysis. PLoS ONE.

[103] Milone M., Elmore U., Vignali A., Gennarelli N., Manigrasso M., Burati M., Milone F., De Palma G.D., DelRio P., Rosati R. (2018). Recovery after intracorporeal anastomosis in laparoscopic right hemicolectomy: A systematic review and meta-analysis. Langenbeck’s Arch. Surg..

[104] Sato K., Inomata M., Kakisako K., Shiraishi N., Adachi Y., Kitano S. (2003). Surgical technique influences bowel function after low anterior resection and sigmoid colectomy. Hepatogastroenterology.

[105] Papanikolaou I.G. (2014). Robotic surgery for colorectal cancer: Systematic review of the literature. Surg. Laparosc. Endosc. Percutaneous Tech..

[106] Wong D.J., Wong M.J., Choi G.H., Wu Y.M., Lai P.B., Goh B.K.P. (2019). Systematic review and meta-analysis of robotic versus open hepatectomy. ANZ J. Surg..

[107] Advincula A.P., Song A. (2007). The role of robotic surgery in gynecology. Curr. Opin. Obstet. Gynecol..

[108] Boylu U., Oommen M., Raynor M., Lee B.R., Thomas R. (2010). Robot-assisted laparoscopic radical prostatectomy in patients with previous abdominal surgery: A novel laparoscopic adhesiolysis technique. J. Endourol..

[109] Petros F.G., Patel M.N., Kheterpal E., Siddiqui S., Ross J., Bhandari A., Diaz M., Menon M., Rogers C.G. (2011). Robotic partial nephrectomy in the setting of prior abdominal surgery. BJU Int..

[110] Gkegkes I.D., Mamais I.A., Iavazzo C. (2017). Robotics in general surgery: A systematic cost assessment. J. Minim. Access Surg..

