# Supplementary figures and images for: The Role of Robotic Visceral Surgery in Patients with Adhesions: A Systematic Review and Meta-Analysis
Source: J Pers Med. 2022 Feb 18;12(2):307. doi: 10.3390/jpm12020307 (PMC8878352; doi:10.3390/jpm12020307)

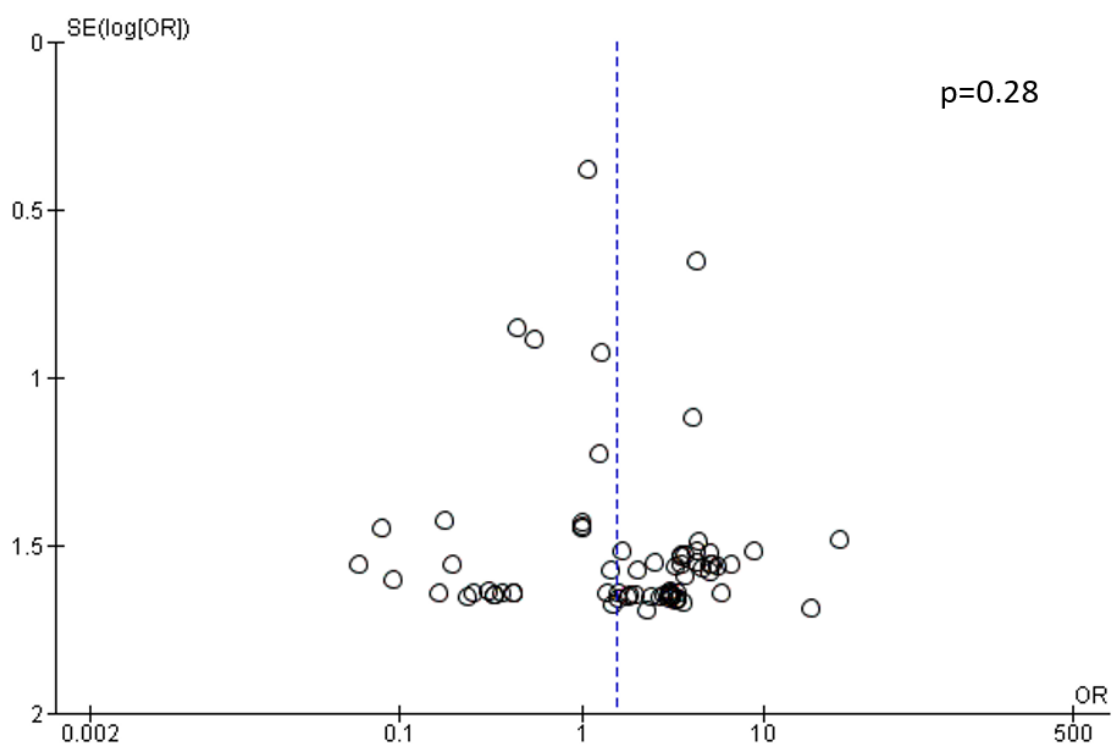

Figure S1. Forest plot analysis of the included studies.

Supplement: Supplementary file 1 [file jpm-12-00307-s001.zip › jpm-1515527-supplementary.pdf]
